# An Anti-Human Thymocyte Globulin-Based Reduced-Intensity Conditioning Regimen Is Associated with a Higher Quality of Life and Lower Organ Toxicity without Affecting Lymphocyte Reconstitution

**DOI:** 10.1371/journal.pone.0073755

**Published:** 2013-09-09

**Authors:** Zheng-Ping Yu, Jia-Hua Ding, Bao-An Chen, Yu-Feng Li, Bang-He Ding, Jun Qian

**Affiliations:** 1 Department of Hematology (Key Department of Jiangsu Medicine), ZhongDa Hospital, Southeast University, Nanjing, China; 2 Hematology Division, Huaian Hospital, Nanjing Medical University, Huaian, China; 3 Hematology Division, Zhenjiang Hospital, Zhenjiang Medical University, Zhenjiang, China; B.C. Cancer Agency, Canada

## Abstract

Reduced-intensity (RIT) conditioning regimens are gaining increased attention as a result of their advantages and efficacy. However, no data are available regarding whether these regimens improve patient quality of life (QoL). In our study, health-related QoL (HRQoL) was retrospectively assessed in 111 patients with hematological malignancies. Analysis of the Quality of Life Questionnaire indicated that 35 of the RIT patients were able to perform their normal work and returned to their baseline levels of function 2 to 3 months after transplantation. In the myeloablative (MA) group, only 24 patients were able to resume work, and these patients returned to their baseline levels of function 6 to 8 months after transplantation (68.6% vs. 40.0%, *P* = 0.004). Grade III–IV organ toxicity occurred in 20% of the RIT patients and in 52% of the MA patients (*P* = 0.001), and the cumulative incidences of grades III–IV acute graft-versus-host disease (GVHD) were 13.7% and 35.0% in RIT and MA patients, respectively (*P* = 0.015). In conclusion, the RIT conditioning regimens were well tolerated by the patients, with a low incidence of transplant-related mortality (TRM) and serious acute GVHD. In addition, these regimens minimized procedure-related toxicity, improved QoL and did not influence lymphocyte reconstitution; however, OS was similar for both regimens because the relapse rate was relatively increased in the RIT groups.

## Introduction

The conventional wisdom in allogeneic hematopoietic stem cell transplantation (allo-HSCT) is that one should use large doses of radiotherapy and chemotherapy to maximize the killing of tumor cells in the body, to promote engraftment, to reduce GVHD and to prevent the recurrence of the primary disease after transplantation; however, the combination of high dose radiotherapy and chemotherapy results in severe bleeding, infection, vital organ damage and other complications, which often endanger patients' lives [1], [2]. Thus, this measure is limited in elderly patients and younger patients in a poor general condition, but it still has a higher transplant-related mortality (TRM) [3]. In recent years, people have come to realize that the efficacy of allo-HSCT depends primarily on the post-transplant graft-versus-leukemia (GVL) effect of the graft in the body of the recipient rather than on a high dose of radiotherapy or chemotherapy [4]. Based on this realization, reduced-intensity conditioning (RIT) has evolved; this therapeutic approach emphasizes a strong inhibition of the recipient's immune function rather than trying to destroy the bone marrow to promote engraftment [5]–[7]. Compared with traditional myeloablative (MA) transplantation, RIT has a wide range of applications, lower organ toxicity and fewer transplant-related complications [8]–[10]. However, no data regarding how these regimens improve patient quality of life (QoL) are available. In this study, we have developed a conditioning regimen that combines fludarabine and busulfan (FB) with a low dose of ATG as an adjuvant treatment to improve patient QoL after allo-HSCT. We compared the outcomes of HSCT in patients following these two regimens and demonstrated that these regimens were associated with significant differences in outcomes, including organ toxicity, QoL, GVHD, relapse and TRM after HSCT. However, there was no significant difference in the overall survival (OS) or lymphocyte reconstitution between these regimens.

## Patients and Methods

### Patients

A total of 111 consecutive HSCT patients with hematological malignancies were treated with either an MA regimen or a low dose ATG-based RIT regimen at 3 transplantation centers. All of the patients included in this study had one or more of the following risk factors: acute leukemia in CR_1_ with a high risk of relapse (with a genetic indicator for a poor prognosis or complex chromosomal abnormalities), acute leukemia in CR_2_ or above, acute leukemia in relapse failing to achieve remission, chronic myeloid leukemia (CML) in the chronic phase (CP) for up to 2 years or a white blood cell (WBC) count of more than 100×10^9^/l or CML in the accelerated phase. At the 3 transplantation centers, a total of 51 patients received a low dose of ATG-based RIT, and 60 patients received conventional MA conditioning (control group) during the same period. The assignment of patients to a particular treatment group was a clinical decision. All of the patients consented to the use of medical information for this research, and the Southeast University, Nanjing Medical University and Zhenjiang Medical University ethics committees approved the study and required the participants to sign an informed consent form. The transplantations were performed between July 2005 and September 2010. The data of the MA and RIT patients through October 2011 were compared and analyzed, and the characteristics of the patients and donors are displayed in **Table** **1**.

**Table 1:** Characteristics of patients, according to myeloablative (MA) and Reduced intensity (RIT) conditioning regimens.

Characteristic	Conditioning regimen		
	RIT	MA	p-value
Number of patients	51	60	
Median age (range)	41(15∼58)	38(13∼51)	0.652
Sex, M/F	28/23	32/28	0.510
DiagnosisAMLALLCML	10932	171726	0.123
Donor and HLAhistocompatibility No.Related 6/6 identity1/6 mismatch2/6 mismatchUnrelated 10/10 identity1/10 mismatch	1912893	171410145	0.230
ABO compatibility – No.MatchedMinor mismatchMajor mismatch	211218	311415	0.442
Disease activityActiveRemission	2724	3327	0.490

Activity disease: chronic myeloid leukemia in accelerated phase, acute leukemia was more than two induction therapies with residual leukemia cells or failed to achieve remission.

### Conditioning regimens and GVHD prophylaxis

The patients in the MA group received modified busulfan and cyclophosphamide (BUCY) treatment [11] as the conditioning regimen. Cytarabine (2 g/m^2^) was administered intravenously every 12 h for a total of 2 days, and a dose of busulfan (1 mg/kg) was administered every 6 h for a total of 16 doses. Cyclophosphamide (60 mg/kg/day) was administered intravenously for 2 days following the completion of the busulfan treatment, followed by semustine (MeCCNU, 250 mg/m^2^) in a single oral dose. Patients in the RIT group were administered fludarabine (30 mg/m^2^ on days -6 to -2) combined with busulfan (0.8 mg/kg, administered every 6 h for a total of 10 doses). Patients in the RIT group were then administered 5 mg/kg anti-thymocyte globulin (ATG, Fresenius AG, Oberursel, Germany) on days -2 to -1, for a total dose of 500-600 mg. The protocols were the same at all three institutions. All of the patients received cyclosporine A (CsA) and a short course of methotrexate (MTX) as prophylaxis against GVHD [12], [13]. CsA (2.5 mg/kg/day) was administered via a continuous 24 h intravenous infusion from day -1 to day 28; thereafter, the patient was switched to oral CsA (5 mg/kg, twice per day). During the CsA treatment, the blood concentrations of CsA were maintained between 200 and 400 ng/ml, which were monitored twice per week using a fluorescence polarization immunoassay. The dose of CsA was gradually reduced and discontinued approximately 6 months after transplantation. MTX was administered at a dose of 15 mg/m^2^ intravenously on day 1 after transplantation, followed by 10 mg/m^2^ intravenously on days 3, 6 and 11. The GVHD prophylaxis protocol was similar between the two regimens.

### Infection prophylaxis and supportive care

All of the patients were hospitalized in rooms with high-efficiency air filters and received standard antibiotics for the prevention of *Pneumocystis carinii* disease with oral trimethoprim-sulfamethoxazole. Ganciclovir (5 mg/kg, twice a day) was intravenously administered for the prevention and treatment of cytomegalovirus (CMV) infection from day -8 before transplantation to day -2 before transplantation [14], [15]. Itraconazole was administered intravenously to patients with a mycotic infection [16]. Prostaglandin E1 (20 µg/d once daily) was administered intravenously for the prophylaxis of hepatic veno-occlusive disease (VOD) [17]. The patients received transfusions when the platelet level was below 20×10^9^/l or the hemoglobin level decreased to 70 g/l during hospitalization; all blood products were irradiated. All regimen-related toxicities were graded and recorded according to the National Cancer Institute (NCI) common toxicity criteria [18].

### Evaluation of quality of life

The European Organization for Research and Treatment of Cancer Quality of Life Questionnaire (EORTC QLQ C30, version 1.0) was used as an effective tool to assess the HRQoL of the cancer patients [19]. The QoL evaluation of all the patients was performed according to the five functional scales and an overall health/global quality of life scale. Patients who achieved high scores had better function. Three symptom scales were used to measure fatigue, pain and nausea and vomiting, and six single items were used to assess the symptoms commonly reported in cancer patients (Table 2). Higher scores on the symptom scales and single items indicated greater symptomatology or obstacles. The mean scale and item scores were transformed into a 0–100 scale as described in the EORTC Scoring Manual [20]. All of the patients were evaluated at four time points (baseline and follow-up at 3, 6 and 12 months after transplantation). OS was calculated from the date of the transplantation to the last follow-up date or the date of death.

**Table 2:** HRQOL at MA or RIT groups after hematopoietic stem cell transplantation.

	Myeloablative (95% CI)	Reduced intensity (95% CI)
	(n = 60)	(n = 51)
Functioning scales^a^		
Cognitive	74(69–81)^b^	89(82–95)
Emotional	70(65–76)	80(75–86)
Physical	58(54–67)	87(81–94)
Role	61(54–69)	79(69–88)
Social	52(48–61)	64(55–73)
Global QoL	55(49–61)^c^	79(72–85)
Symptom scales^d^		
Fatigue	44(37–51)^c^	21(18–34)
Nausea/vomiting	13 (8–21)	11(7–16)
Pain	19(14–25)	13 (6–19)
Single items^d^		
Appetite loss	14(6–21)	9(3–14)
Constipation	15(8–24)	4 (1–7)
Diarrhea	19(9–28)	12(4–20)
Dyspnea	29(19–40)	12(6–19)
Economic impact	12(5–21)	14(6–23)
Sleep disturbance	28(21–39)	13(6–21)

a Higher scores indicate better functioning.

b Mean (95% confidence interval).

c p<0.01 for differences in mean scores, MA vs RIT.

d Higher scores indicate more symptomatology.

### Lymphocyte immune-reconstitution studies

We collected peripheral blood samples of patients into 1.5–2 ml of EDTA-K2 anticoagulant and analyzed CD3+ lymphocytes, CD4+ lymphocytes, CD8+ lymphocytes, CD19+ lymphocytes and CD16+/CD56+ (NK) cellular expression levels using flow cytometry (fluorescence-activated cell sorting Vantage cytometers, BD Biosciences). The analysis of lymphocyte recovery was performed at the following five time points: pre-transplant and 3, 6, 9 and 12 months post-transplant.

### Clinical endpoints and statistical analysis

The primary endpoints of the analysis were treatment-related organ toxicity and mortality and the incidence and severity of GVHD. The secondary endpoint of the study was overall survival (OS) time, defined as the start of treatment to the time of death from any cause. Engraftment was defined as an absolute neutrophil count (ANC) of >0.5×10^9^/l for the first 3 consecutive days and a platelet count of >20×10^9^/l for at least 7 days independent of a transfusion. The follow-up assessment was performed with the cytogenetic analysis of bone marrow aspirates at 30, 60, 90 and 180 days after transplantation. In addition, chimerism was identified with the analysis of short tandem repeat polymorphisms using PCR (STR-PCR) in gender-matched transplantations and with chromosomal analysis using fluorescent in situ hybridization (FISH) in gender-mismatched transplantations [21].

Continuous variables were described as the mean ± SD with the median range in parentheses. A log-rank test was applied for comparisons between subsets. The cumulative incidence of GVHD and the OS rate were analyzed using the Kaplan-Meier method [22]. Univariate and multivariate analyses were performed using the Cox proportional hazard model to determine these outcomes. All values were two tailed, and p<0.05 was considered statistically significant. A statistical software package (SPSS 11.5) was used for all of the analyses [23].

## Results

Engraftment was observed in all of the patients, and the median times to an ANC of 0.5×10^9^/l and a platelet count of 20×10^9^/l in the MA group were 13 (range, 8–20) and 14 (range, 7–39) days, respectively. The median times to ANC and platelet recovery in the RIT group were 14 (range, 10–22) and 16 (range, 9–42) days, respectively. There was no significant difference between the MA and RIT groups for this parameter (*P*>0.05). Full donor-type chimerism (90%) was observed at 30 days, and follow-up observations for chimerism occurred at 60, 90 and 180 days after transplantation. In the RIT group, seven patients presented mixed chimerism for 3–4 months after transplantation and then converted to full donor type after being given a donor lymphocyte infusion (DLI), two CML patients with disease recurrence achieved complete remission after a DLI, one patient with acute myeloid leukemia (AML) relapsed and improved, and two patients with acute lymphocytic leukemia (ALL) relapsed and died after receiving a DLI.

### Regimen-related toxicities, GVHD and TRM

**Figure 1:**
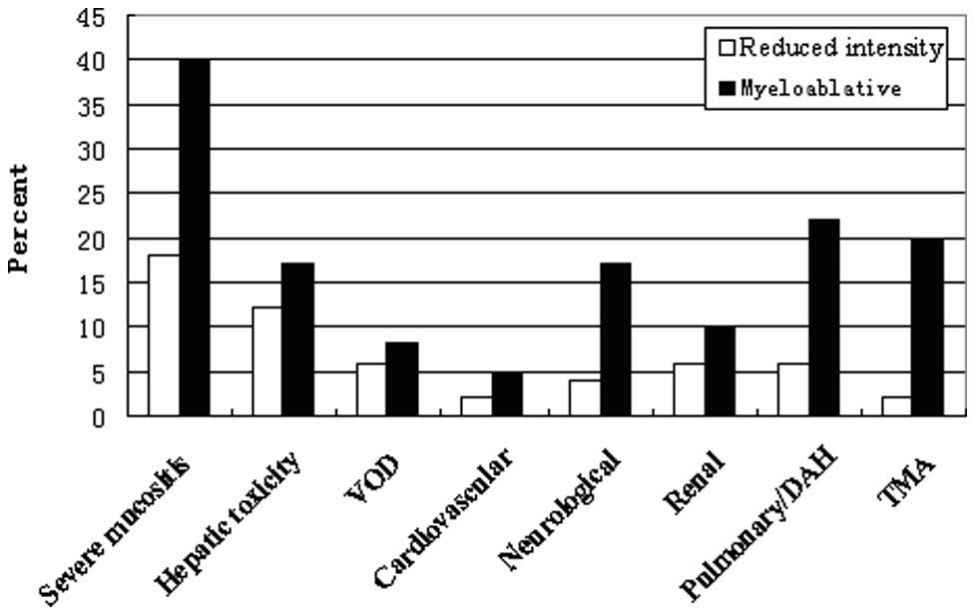
Distribution of individual comorbidities among reduced intensity regimens and myeloablative patients as assessed by the NCI.

According to the NCI organ toxicity grading system, a total of 41 patients had grade III–IV organ toxicity: 10 (20%) from the RIT group and 31 (52%) from the MA group (P = 0.001). Mucositis, neurological disease, pulmonary disease and transplantation-associated thrombotic microangiopathy were more common in the MA group (**Figure 1**).

Among the 111 patients, 59 (53.2%) developed grade I­IV acute GVHD after transplantation; grade III–IV acute GVHD developed in 7 of 51 patients who received RIT and in 21 of 60 patients who received MA, and the cumulative incidences were 13.7% and 35.0%, respectively (*P* = 0.015). One patient in the RIT group and seven patients in the MA group died of severe acute GVHD. Among all 60 patients administered the MA regimen, 24 developed chronic GVHD, 9 of whom developed extensive cGVHD. In the RIT group, 18 patients developed cGVHD, including 6 patients with extensive disease and 12 patients with limited disease (**Table** **3**).

**Table 3:** Engraftments and GVHD in RIT and MA conditioning regimens.

Index	RIT (n = 51)	MA (n = 60)	p
Median days (range) to neutrophil	14 (10∼22)	14 (10∼22)	0.52
engraftment >0.5×10^9^/l			
Median days (range) to platelet	16 (9∼42)	14 (7∼39)	0.55
engraftment>20×10^9^/l			
GVHD			
Acute, grades III– IV (%)	7/51 (13.7%)	21/60 (35.0%)	0.015
Chronic GVHD (%)	18/51 (35.3%)	24/60 (40.0%)	0.696
Limit	12/51 (23.5%)	15/60 (25.0%)	
Extensive	6/51 (11.8%)	9/60 (15.0%)	

In the MA group, TRM occurred in 22 patients, with a cumulative incidence of 36.7% at four years after the HSCT. The causes of death included organ toxicity (n = 7), infections (n = 8) and complications from serious aGVHD (n = 7). There was a significant difference in the cumulative incidence of TRM among patients who received MA compared to patients who received RIT (36.7% vs. 11.8%, *P* = 0.004). The univariate analysis indicated that the type of disease, status of the disease, conditioning regimen and organ toxicity influenced the risk of TRM. Age, gender and having an unrelated donor were not identified as risk factors for TRM. A multivariate analysis indicated that the most significant risk factors for TRM were conditioning with MA, organ toxicity, active disease and a diagnosis of ALL, with hazard ratios of 2.14, 2.51, 2.06 and 2.63, respectively (**Table** **4**).

**Table 4:** Analysis of factors predicting for transplant-related mortality after HSCT/

Category	*No*	TRM(*No*)	p-value	HR(p-value)	
All patients	111	28			
Age(years)					
<40	35	11	0.502		
≥40	76	17			
Gender					
Male	62	19	0.289		
Female	49	9			
Donor					
Sibling	80	16	0.169		
MUD	31	12			
Disease					
ALL	26	14	0.006	2.63(1.34–5.25)p = 0.01	
AML	27	8			
CML	58	6			
Status of disease					
Active	60	20	0.048	2.06(1.09–4.38)p = 0.04	
Remission	51	8			
Conditioning					
MA	60	22	0.019		2.14(1.12–4.91)p = 0.04
RIT	51	6			
Organ toxicity					
Yes	41	18	0.011		2.51(1.94–5.69)p = 0.03
No	70	10			

Abbreviations: *No*, Number; HR, hazard ratio with 95% confidence intervals; multivariable analysis, factors with at least borderline statistical significant in the univariant analysis (P<0.1) were included in a Cox proportional hazard model; multivariate analysis (P<0.05) reaching the statistical significance. TRM, transplant -related mortality at 4-years.

### Opportunistic infections

There were 39 cases of opportunistic infections in the patients during the follow-up period. There were 18 cases affecting the respiratory tract, 3 affecting the gastrointestinal tract, 4 affecting the urinary tract, 9 affecting the oral mucosa, 3 affecting the perianal soft tissue and 2 with a fever and unclear foci. A total of 25 patients in the MA group were infected, including 8 infections that caused respiratory failure and death. A total of 14 patients in the RIT group were infected, including 3 who died of severe infections. However, there was no significant difference in the incidence of infections between the MA and RIT groups (41.7% vs. 27.5%, respectively; *P* = 0.162).

### Health-related quality of life

After the allo-HSCT, there were statistically significant differences at baseline, trending toward a better QoL in the RIT group as assessed using the nine scales and six single items. The differences in global QoL (79 [95% confidence interval (CI) 72–85] vs. 55 [CI 49–61]), physical (87 [CI 81–94] vs. 58 [CI 54–67]) and fatigue (21 [CI 18–34] vs. 44 [CI 37–51]) were the most pronounced. The symptom scores of the RIT group were similar to those of the MA group in nausea/vomiting and pain, whereas the social function and role function scores were significantly improved in the RIT group (**Table 2**). The RIT patients generally reported smaller changes from their baseline scores during the first year compared to those of the MA patients and exhibited a more rapid return to normal baseline levels. At 2 months post-transplantation, the social and role function scores were similar to or greater than the baseline scores, as was the global QoL after 3 months. Most symptom scores and single items had returned to baseline levels after 2 months, except for dyspnea and fatigue. A continuous improvement was observed moving toward the 1-year assessment, suggesting that the most pronounced clinically significant improvements occurred within the first year. Furthermore, our data suggest that the MA patients recovered more slowly from the transplantation. The MA patients had a more dramatic decline in QoL and returned to baseline levels 6 to 8 months after transplantation, whereas the RIT patients recovered to baseline levels of function 2 to 3 months after transplantation. Notably, six months after transplantation, the MA patients were more likely to have visited a physician within the past month, including eight patients who were still on medication, seven patients who required occasional assistance and were unable to care for most of their personal needs, and ten patients who were able to take care of themselves but could not participate in normal activities; only 24 patients were able to return to work, school or housework. In the RIT group, 10 patients were able to live at home and take care of themselves, and 35 patients from this group were able to perform their normal daily activities and return to work. There was a significant difference in the recovery of baseline levels of function in QoL between the RIT and MA groups (68.6% vs. 40.0%, *P* = 0.004).

### Lymphocyte reconstitution in all patients

**Figure 2:**
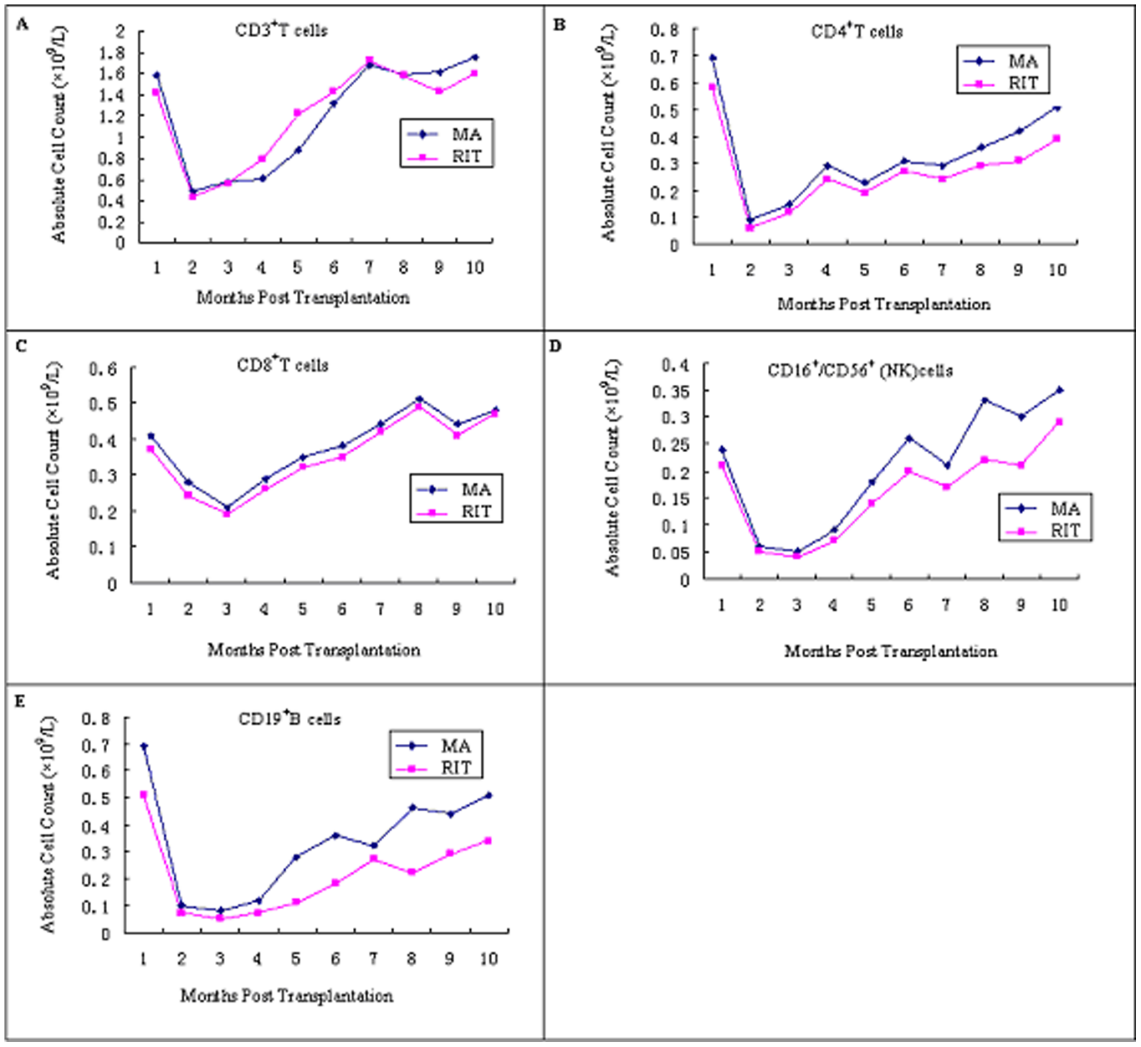
Comparative outcome of 5 lymphocyte subsets reconstitution in the recovery of baseline levels of time between the RIT and MA groups.

A decline in CD4+ and CDl9+ lymphocytes was most obvious. In the RIT group, CD4+ lymphocytes recovered to near normal levels within a mean of 16 months post-transplant; these lymphocytes in the MA group returned to normal levels within a mean of 12 months after transplantation. After transplantation, CDl9+ cells required a mean of 14 to 18 months before the counts reached normal levels. In contrast, the CD8+ cell counts had rapid recovery times of 4 to 8 months and 3 to 6 months post-transplant in the RIT and MA groups, respectively. CD3+ cell counts achieved normal levels within 3 to 4 months after transplantation. CD16+/CD56+ cell counts reached normal levels within 1 to 2 months or were even above normal levels 3 months after transplantation. We analyzed 5 lymphocyte subsets, namely CD3+, CD4+, CD8+ and CD19+ lymphocytes and NK cells; there was no statistically significant difference in the recovery of baseline levels or the time to lymphocyte reconstitution between the RIT and MA groups (***P*** **>0.05,** **Figure 2**).

### Relapse and survival

With a median follow-up of 42 months (range, 2–62 months), 68 patients survived, and 43 died. Twenty-eight patients died of treatment-related complications, and 15 died of disease relapse after transplantation, including 6 patients who had acute leukemia in CR_1_ with a high risk of relapse, 4 patients who had acute leukemia in CR_2_, 2 patients who failed to achieve remission, and 3 patients who had CML in the accelerated phase. Furthermore, 3 of the 15 patients were from the MA group. The 4-year probability of relapse was 5% in the MA group and 23.5% in the RIT group. In October 2011, 35 MA patients were alive, and the disease OS was 58.3%. In the RIT group, 12 patients died from recurrent leukemia, and 6 patients died from transplant-related complications; the disease OS was 64.7%. The causes of transplantation-related death included one case of GVHD, three cases of infection and two cases of organ toxicity. The Kaplan–Meier estimate of OS in the patients who underwent allo-HSCT was not significantly different between the MA and RIT groups (58.3% vs. 64.7%, respectively; *P* = 0.560).

## Discussion

RIT conditioning regimens in allo-HSCT are characterized by reducing the radiotherapy and chemotherapy doses in the pretreatment program and strengthening the immune suppression before the transplant to reduce transplant-related toxicity and to expand the applications of transplantation [24]–[26]. Replacing high-dose MA therapy with a low-dose, ATG-based RIT conditioning regimen potentially enables the treatment of patients previously considered too old for allo-HSCT or patients who have accompanying organ dysfunction or are ineligible for conventional allo-HSCT.

Evidence in the literature suggests that an RIT conditioning regimen is a standard procedure to prevent or reduce organ toxicity, graft rejection, GVHD and TRM [27]. In addition to these outcomes, QoL is increasingly being used to assess the success of various therapies. In a cross-sectional study, Bieri et al. [28] evaluated the QoL in 124 adult patients in remission after allogeneic HSCT. The assessment of QoL was conducted using two questionnaires: the EORTC QLQ-C30 and the Functional Assessment of Cancer Therapy (FACT). The measures analysis revealed that patient age at the time of HSCT and employment status were significantly associated with QoL, whereas other factors, including disease and disease stage, did not impact patient QoL. Campelo MD et al. [29] examined the QoL effects in 47 patients undergoing RIT allo-HSCT and compared these with a similar subgroup of patients receiving autologous stem cell transplantation. There was no significant difference between the two groups for any of the single items within functional, social/role, psychological distress or satisfaction. However, little has been reported in the published literature regarding whether low-dose ATG-based RIT regimens improve QoL. In our study, the analysis indicated that 35 patients were able to perform their normal work and returned to their baseline levels of function 2 to 3 months after transplantation. In the MA group, only 24 patients were able to resume work, school or housework, and these patients returned to their baseline levels of function 6 to 8 months after transplantation. In summary, these data suggest that people in the low-dose ATG-based RIT group have a better QoL. It is known that GVHD has a major negative impact on the QoL of long-term survivors after allo-HSCT [30]. However, the low-dose ATG-based RIT conditioning regimen appeared to be a very efficient strategy to improve the QoL in our study, which is consistent with previous reports that demonstrated that the use of an RIT regimen had a low incidence of GVHD and organ toxicity [27], [31].

ATG is a polyclonal antibody with a variety of immunomodulatory effects, including the ability to remove T lymphocytes and its variety of effects on the immune system. Thus, ATG affects multiple immune regulatory pathways, and the inclusion of such immune suppression therapy helps to reduce organ rejection and the occurrence of GVHD [32]. However, high doses of ATG will reduce the GVL effect and delay immune reconstitution, which will increase the risk of infection [33]. In this study, we demonstrated that a low-dose ATG-based RIT regimen was well tolerated and was associated with low TRM without affecting lymphocyte reconstitution or the GVL effect. This regimen also resulted in a reduction in the incidence of severe and hazardous acute GVHD and improvements in QoL. Moreover, in contrast to the MA patients, we did not observe a high incidence of complications due to infection in the RIT patients.

Most studies suggest that T lymphocyte immunity plays an important role in the anti-tumor effect and GVHD after transplantation. Among these lymphocytes, CD4+ T cells primarily mediate graft anti-tumor effects, whereas CD8+ T cells play an important role in the occurrence of GVHD [34]. Many factors affect immune reconstitution after transplantation, including recipient age, the source of stem cells, the duration and type of the underlying disease, the occurrence of GVHD, the conditioning regimen and post-transplant immune suppression programs [35], [36]. Our study found that a low-dose ATG-based RIT conditioning regimen and the MA regimen have no statistically significant difference regarding lymphocyte immune function recovery after transplantation. This finding may hold because pretreatment based on a low dose of ATG along with a dose-reduction program resulted in a lower incidence of GVHD after transplantation, and the subsequent reduction in organ toxicity offset the low-dose ATG immune suppression.

We acknowledge that our study involved a limited number of patients with various diseases treated at three transplantation institutions and that some potential bias in the selection of the conditioning regimens may have existed. Therefore, a larger, multicenter, prospective randomized controlled trial is required to confirm our findings and assess the long-term benefits of the low-dose ATG-based, reduced-intensity conditioning approach.
